# The application of metagenomics, radiomics and machine learning for diagnosis of sepsis

**DOI:** 10.3389/fmed.2024.1400166

**Published:** 2024-09-20

**Authors:** Xiefei Hu, Shenshen Zhi, Wenyan Wu, Yang Tao, Yuanyuan Zhang, Lijuan Li, Xun Li, Liyan Pan, Haiping Fan, Wei Li

**Affiliations:** ^1^Clinical Laboratory, Chongqing University Central Hospital, School of Medicine, Chongqing University, Chongqing, China; ^2^Chongqing Key Laboratory of Emergency Medicine, Chongqing Emergency Medical Center, Chongqing, China; ^3^Department of Blood Transfusion, Chongqing University Central Hospital, School of Medicine, Chongqing University, Chongqing, China; ^4^Intensive Care Unit, Chongqing University Central Hospital, School of Medicine, Chongqing University, Chongqing, China

**Keywords:** sepsis, metagenomics, blood test indicators, radiomics, machine learning

## Abstract

**Introduction:**

Sepsis poses a serious threat to individual life and health. Early and accessible diagnosis and targeted treatment are crucial. This study aims to explore the relationship between microbes, metabolic pathways, and blood test indicators in sepsis patients and develop a machine learning model for clinical diagnosis.

**Methods:**

Blood samples from sepsis patients were sequenced. α-diversity and β-diversity analyses were performed to compare the microbial diversity between the sepsis group and the normal group. Correlation analysis was conducted on microbes, metabolic pathways, and blood test indicators. In addition, a model was developed based on medical records and radiomic features using machine learning algorithms.

**Results:**

The results of α-diversity and β-diversity analyses showed that the microbial diversity of sepsis group was significantly higher than that of normal group (*p* < 0.05). The top 10 microbial abundances in the sepsis and normal groups were Vitis vinifera, *Mycobacterium canettii, Solanum pennellii, Ralstonia insidiosa, Ananas comosus, Moraxella osloensis, Escherichia coli, Staphylococcus hominis, Camelina sativa*, and *Cutibacterium acnes*. The enriched metabolic pathways mainly included Protein families: genetic information processing, Translation, Protein families: signaling and cellular processes, and Unclassified: genetic information processing. The correlation analysis revealed a significant positive correlation (*p* < 0.05) between IL-6 and Membrane transport. Metabolism of other amino acids showed a significant positive correlation (*p* < 0.05) with *Cutibacterium acnes, Ralstonia insidiosa, Moraxella osloensis*, and *Staphylococcus hominis*. Ananas comosus showed a significant positive correlation (*p* < 0.05) with Poorly characterized and Unclassified: metabolism. Blood test-related indicators showed a significant negative correlation (*p* < 0.05) with microorganisms. Logistic regression (LR) was used as the optimal model in six machine learning models based on medical records and radiomic features. The nomogram, calibration curves, and AUC values demonstrated that LR performed best for prediction.

**Discussion:**

This study provides insights into the relationship between microbes, metabolic pathways, and blood test indicators in sepsis. The developed machine learning model shows potential for aiding in clinical diagnosis. However, further research is needed to validate and improve the model.

## 1 Introduction

Sepsis is a severe organ dysfunction endangering life, resulting from impaired host function triggered by infection (1, 2). Epidemiological survey data show (3) that sepsis is characterized by a high incidence and mortality, and its incidence has been increasing in recent years. Each year, sepsis impacts over 30 million individuals globally and leads to around 6 million deaths (4). According to domestic statistics, around 20.6% of ICU patients experience sepsis, the 90-day overall mortality is 35.5%, and the rate is as high as 51.94% combined with septic shock (5). Sepsis is a serious threat to physical health.

Sepsis has a variety of clinical manifestations, including fever, increased heart rate, shortness of breath, hypotension, changes in consciousness, etc. (6). Additionally, patients may experience systemic multiple organ dysfunction such as pneumonia, acute respiratory distress syndrome (ARDS), renal impairment, and cardiac insufficiency. Severe sepsis can lead to shock and even death. Therefore, an early diagnosis is particularly important for the treatment of sepsis. It is diagnosed based on the evidence of infection and manifestations of systemic inflammation (7). Currently, the diagnosis of sepsis is mainly based on blood culture (8), and white blood cell (WBC) count, classification, C-reactive protein (CRP), and procalcitonin precursor (PCT) are determined for auxiliary diagnosis (9).

With the advancement of big data analysis, genomics research, and biomarker research, the pathogenesis of sepsis will be further clarified (10), which will facilitate molecular biology-oriented diagnosis of sepsis, improve the sensitivity and specificity of diagnosis, and formulate more appropriate diagnostic criteria to reflect the infection and uncontrolled response of the body, thereby further contributing to the early identification and diagnosis of sepsis. It can also reflect the characteristics of dynamic changes in the disease, provide conditions for accurate treatment of sepsis, and improve patient survival. This project aimed to obtain a comprehensive bacterial infectious sepsis-specific pathogenic microorganism through comparative research and whole genome sequencing technology on the metagenomic next-generation sequencing (mNGS) platform, and to establish a prediction model of sepsis integrating radiomics and machine learning algorithms, hoping to provide an implication for its clinical diagnosis.

## 2 Materials and methods

### 2.1 Data analysis

Metagenomic sequencing was performed on 25 patients with sepsis, and nine samples from the normal group were used for metagenomic sequencing. After the raw data were exported, low-quality reads were filtered out and the obtained valid data were used for subsequent analyses. The host sequences were first removed, and sequence alignment was used to infer the species composition and relative abundance of the microbial community, followed by plotting of the species abundance profile. The function, consanguinity, and metabolic pathways of each gene were determined by comparing and annotating the genes to a known database. Through integration and statistical analyses of the annotated results, functional modules and metabolic pathways involved in the microbial community were identified, and their roles in the ecosystem were explored.

### 2.2 Radiomics analysis

The region of interest (ROI)/volume of interest (VOI) usually refers to a lesion that was manually segmented using 3D Slicer v5.1.0. Quantitative features were extracted from the digital images, which were stored in a shared database. The data were mined, and hypotheses were generated or validated. A plugin of the 3D Slicer v5.1.0 software PyRadiomics was used to perform radiomic feature extraction from each ROI. The plugin automatically extracted 851 radioactive features from each ROI. It includes first-order statistical features (energy, entropy, mean, standard deviation, maximum, etc.), shape-and size-based features (maximum 3D diameter, volume, superficial area, etc.), texture features (grayscale co-occurrence matrix GLCM and grayscale run matrix GLRLM), and wavelet-based transform features.

### 2.3 Construction of a machine learning model

In the training set, the selection was made by 10-fold cross-validation and grid search 10 times, and six classification algorithms (LR: logistic regression; RF: random forest; adaboost: adaptive enhancement; SVM: support vector machine; NB: naive Bayes; GBDT: gradient enhancement decision tree) of the corresponding cohort were obtained. The six classification algorithms completed by the training were called to train the data and build the model, and the prediction results of the different models were obtained. The average value of the multiple accuracies was used as the final model score, and the final model was generated simultaneously. A receiver operating characteristic (ROC) curve was plotted for each training model and test result, and the area under the curve (AUC), accuracy, sensitivity, recall rate, and specificity were calculated.

### 2.4 Statistical analysis

Statistical analyses were performed using R software (V4.2.2). The measured data were tested for homogeneity and normality of variance. For measurement data following a normal distribution, the mean ± standard deviation was utilized, with *t*-tests employed for inter-group comparisons. Count data were presented as percentages, and differences between groups were assessed using *χ*^2^ tests, with significance set at *p* < 0.05. Correlation analysis of microorganisms, metabolic pathways, and blood test-related indicators was performed using the Spearman’s correlation coefficient. Based on R software (version 4.0.3) and R studio platform, Lasso feature dimensionality reduction, logistic regression model construction, ROC curve plotting, calibration curve plotting, nomographic chart and clinical decision analysis curve were performed using the corresponding software package.

## 3 Results

### 3.1 Microbiome composition analysis

**Figure 1:**
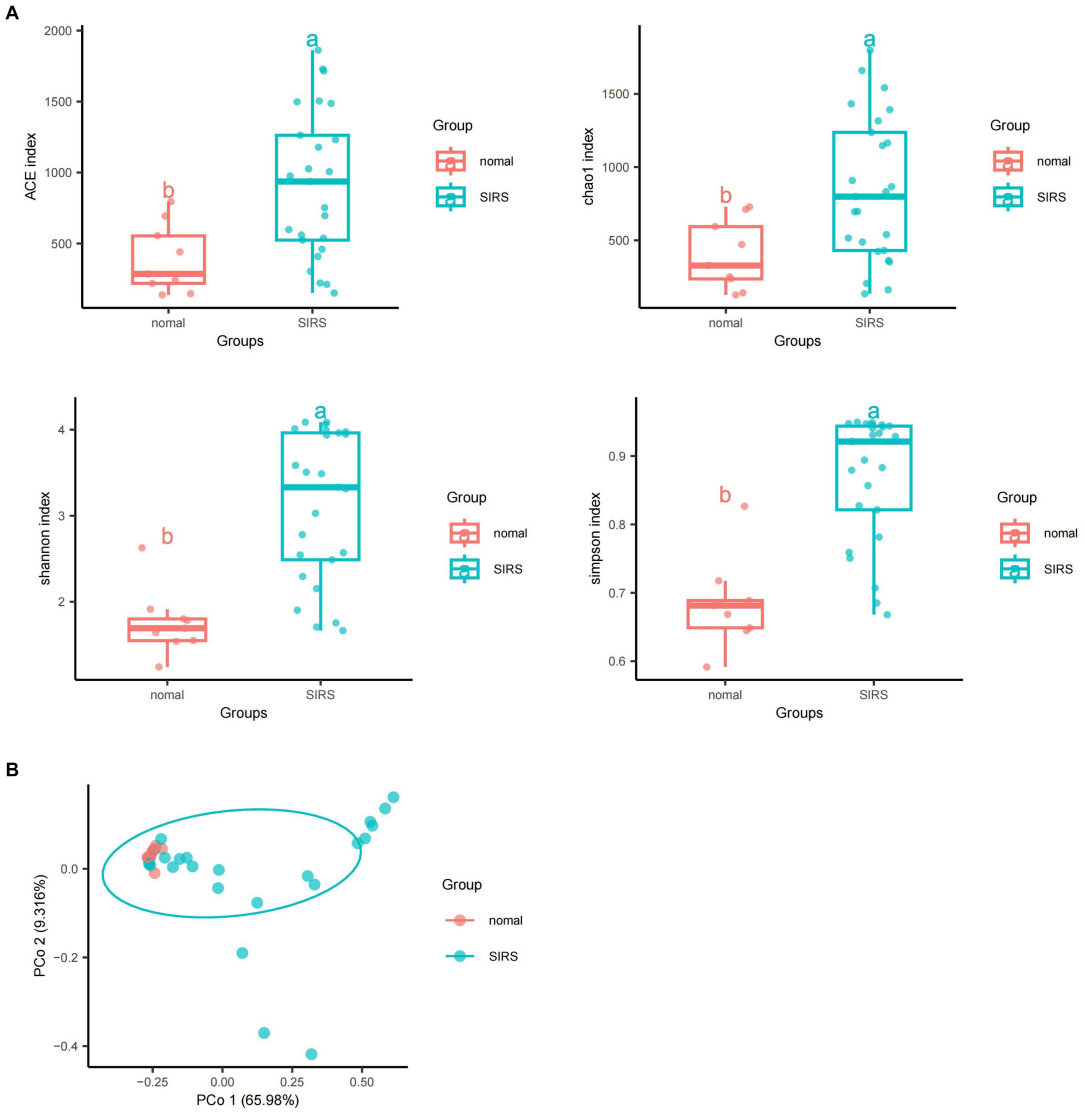
Diversity and abundance analyses of pathogenic microorganisms. **(A)** α-diversity analyses showed that ACE, Chao1, Shannon, and Simpson indices of sepsis patients were significantly higher than those of normal group (*p* < 0.05). **(B)** β-diversity analyses showed that the sample difference between the two groups was small.

**Figure 2:**
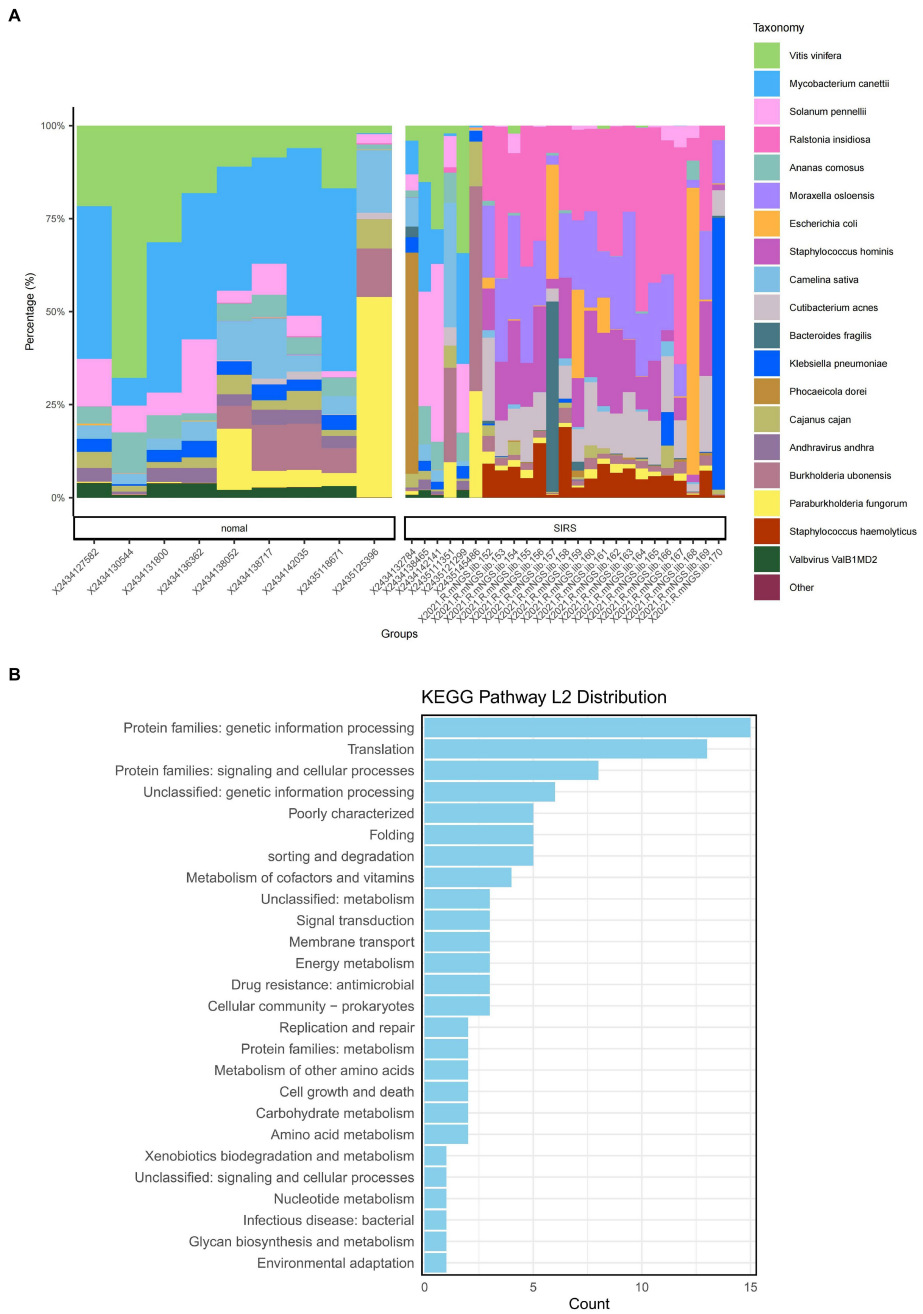
Species annotation results and KEGG metabolic pathway enrichment. **(A)** Bar chart of the relevant abundance of the top 20 species in the sepsis and normal groups. **(B)** Enriched metabolic pathways.

The α-diversity analysis showed that ACE, Chao1, Shannon, and Simpson indices of sepsis patients were significantly higher than those of normal group (*p* < 0.05), indicating that the microbial abundance and diversity of sepsis patients were elevated (Figure 1A). The β-diversity results showed small sample differences between the two groups (Figure 1B). Based on the species annotation results, the top 20 species in terms of abundance were selected at the species level for each sample in the sepsis and normal groups to plot the relative abundance histogram (Figure 2A). The top 10 microbial genera in both groups included *Vitis vinifera*, *Mycobacterium canettii*, *Solanum pennellii*, *Ralstonia insidiosa*, *Ananas comosus*, *Moraxella osloensis*, *Escherichia coli*, *Staphylococcus hominis*, *Camelina sativa*, and *Cutibacterium acnes*. The enriched metabolic pathways were mainly protein families: genetic information processing, translation, protein families: signaling and cellular processes, and unclassified: genetic information processing (Figure 2B).

### 3.2 Correlation analysis

**Figure 3:**
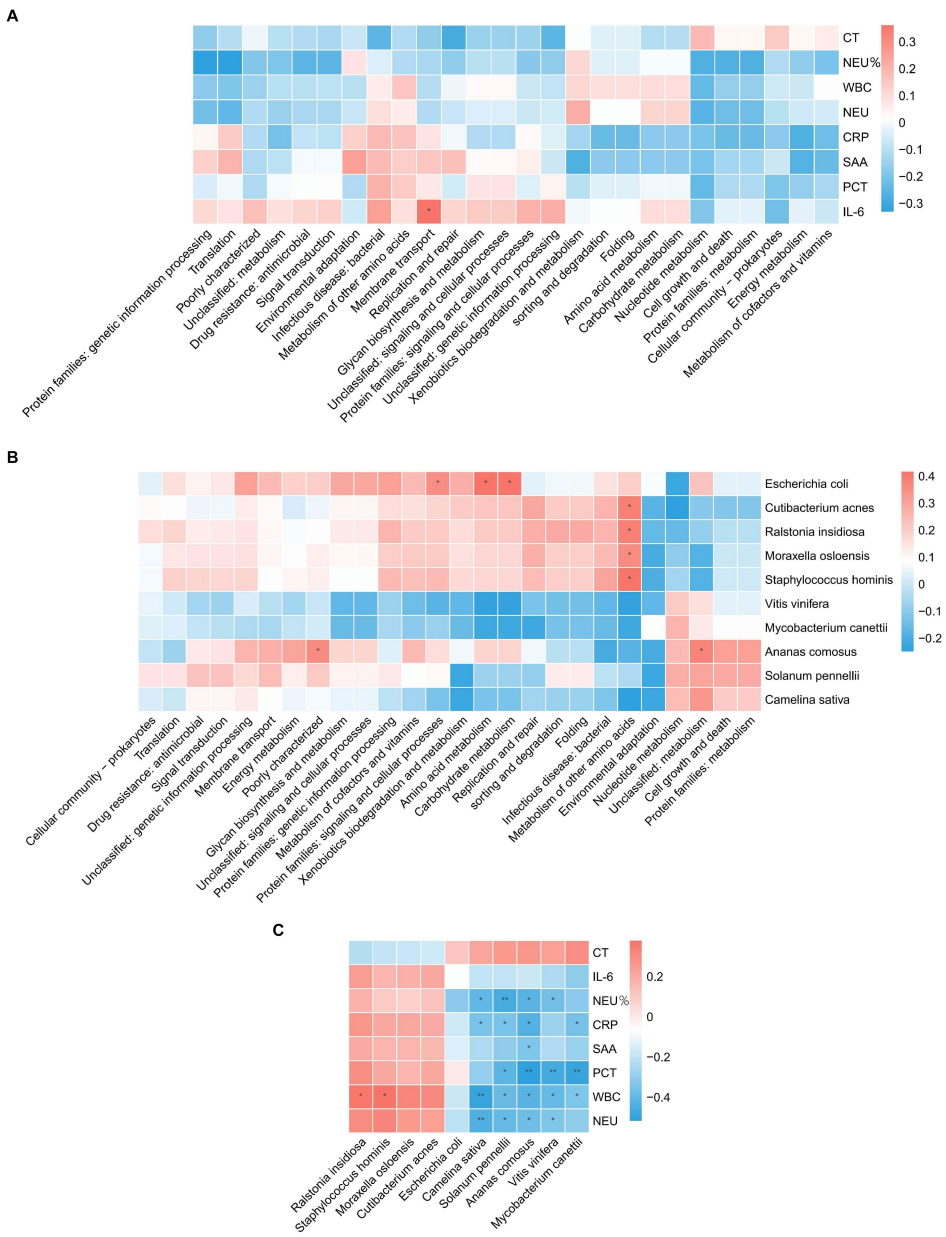
Correlation analysis. **(A)** Correlation between blood test indicators and metabolic pathways. **(B)** Correlation of pathogenic microorganisms and metabolic pathways. **(C)** Correlation between blood test indicators and pathogenic microorganisms.

The analyses revealed (Figures 3A–C) a correlation between metabolic pathways, blood detection indicators, and pathogenic microorganisms, with a significant positive correlation (*p* < 0.05) between IL-6 and membrane transport. Metabolism of other amino acids showed a significant positive correlation (*p* < 0.05) with *Cutibacterium acnes*, *Ralstonia insidiosa*, *Moraxella osloensis*, and *Staphylococcus hominis*. *Ananas comosus* showed a significant positive correlation (*p* < 0.05) with poorly characterized and unclassified: metabolism. *Escherichia coli* showed a significant positive correlation (*p* < 0.05) with protein families: signaling and cellular processes, amino acid metabolism, and carbohydrate metabolism. WBC showed a significant positive correlation (*p* < 0.05) with *Ralstonia insidiosa*, and *Staphylococcus hominis*. *Camelina sativa* showed a significant negative correlation (*p* < 0.05) with NEU%, CRP, WBC, and NEU. *Solanum pennelli* showed a significant negative correlation (*p* < 0.05) with NEU%, CRP, PCT, WBC, and NEU. *Ananas comosus* showed a significant negative correlation (*p* < 0.05) with NEU%, CRP, SAA, PCT, WBC, and NEU. *Vitis vinifera* showed a significant negative correlation (*p* < 0.05) with NEU%, PCT, WBC, and NEU. *Mycobacterium canettii* showed a significant negative correlation (*p* < 0.05) with CRP, PCT, and WBC.

### 3.3 Construction of a machine learning model

**Figure 4:**
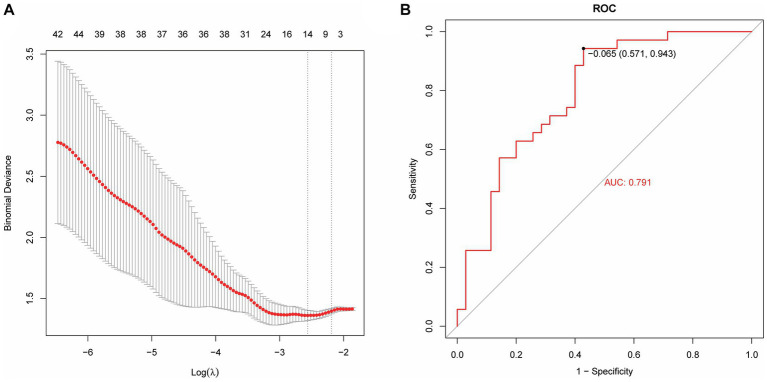
Machine learning model establishment based on radiomics. **(A)** Five important image features screened using LASSO-Cox regression and 10-fold cross validation. **(B)** The AUC value of the model ROC curve is 0.791.

**Figure 5:**
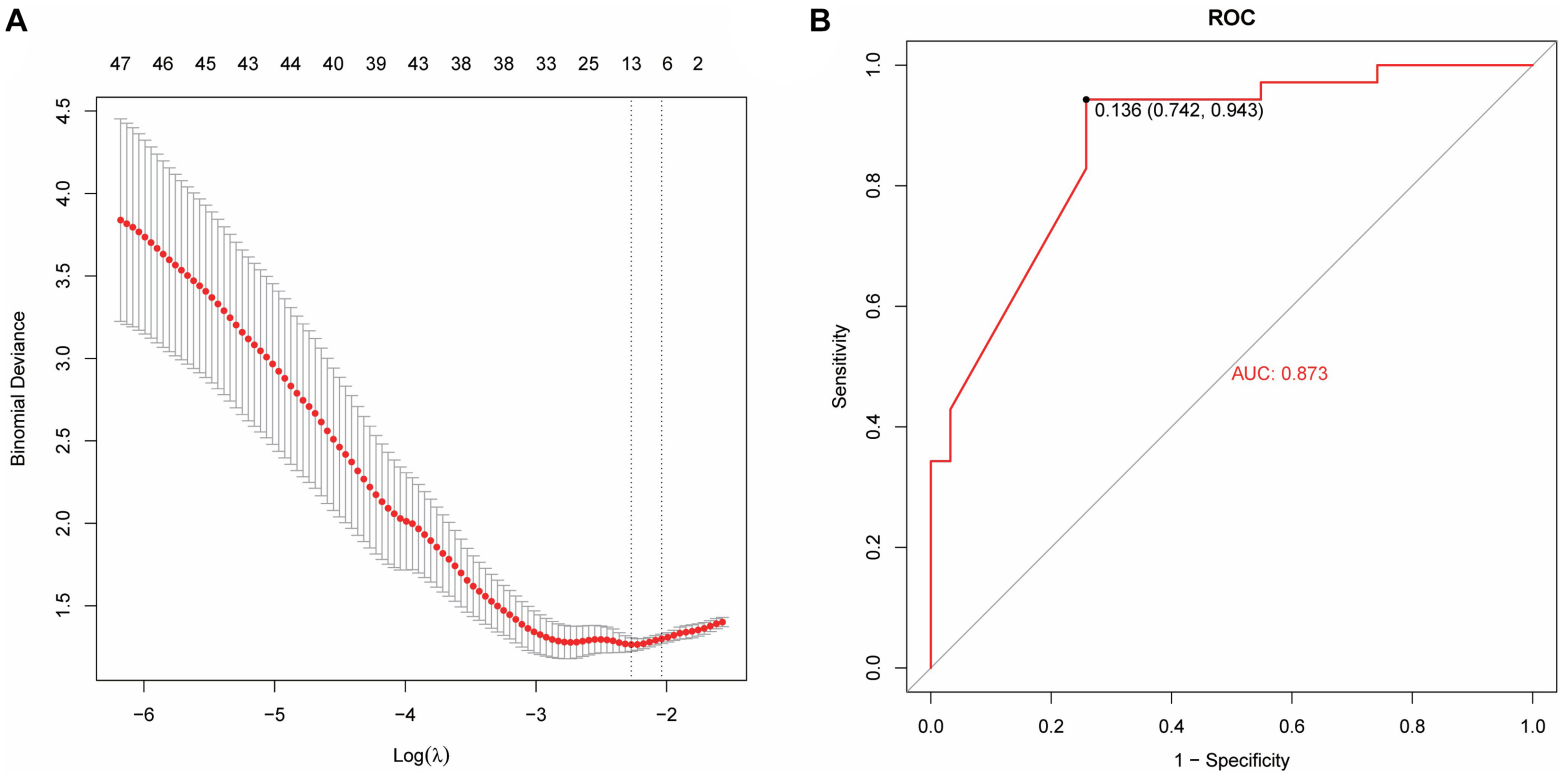
A machine learning model based on medical record data. **(A)** Lasso extraction of seven features from the medical records. **(B)** The AUC value of the model ROC curve is 0.873.

Based on radiomics, five important image features were finally screened using LASSO-Cox regression and 10-fold cross validation including original-shape-sphericity, original-firstorder-10Percentile, wavelet-HHL-glcm-InverseVariance, wavelet-HHH-glszm-ZoneEntropy, and wavelet-LLL-gldm-LargeDependenceLowGrayLevelEmphasis (Figure 4A). Utilizing the previously outlined features, model performance was assessed through the ROC curve, revealing an AUC = 0.791 for the model’s ROC curve (Figure 4B). Significant features were extracted from the medical records using LASSO, including occupancy, inflammation, blood lipids, prognosis, advention, and discharge (Figure 5A). Drawing from the characteristics outlined earlier, the model’s performance was analyzed via the ROC curve, indicating an AUC = 0.873 for the model’s ROC curve (Figure 5B).

**Figure 6:**
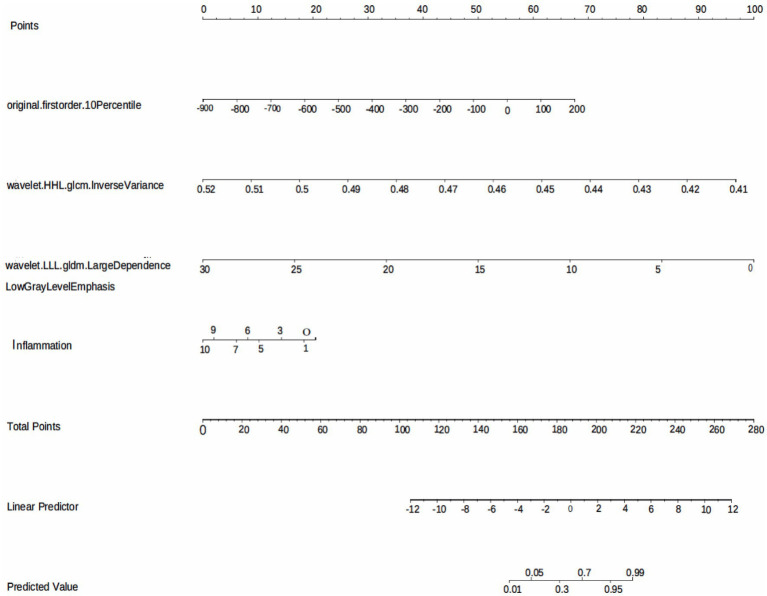
The LR model based on the features extracted from medical records and radiomics is visualized using nomogram.

**Figure 7:**
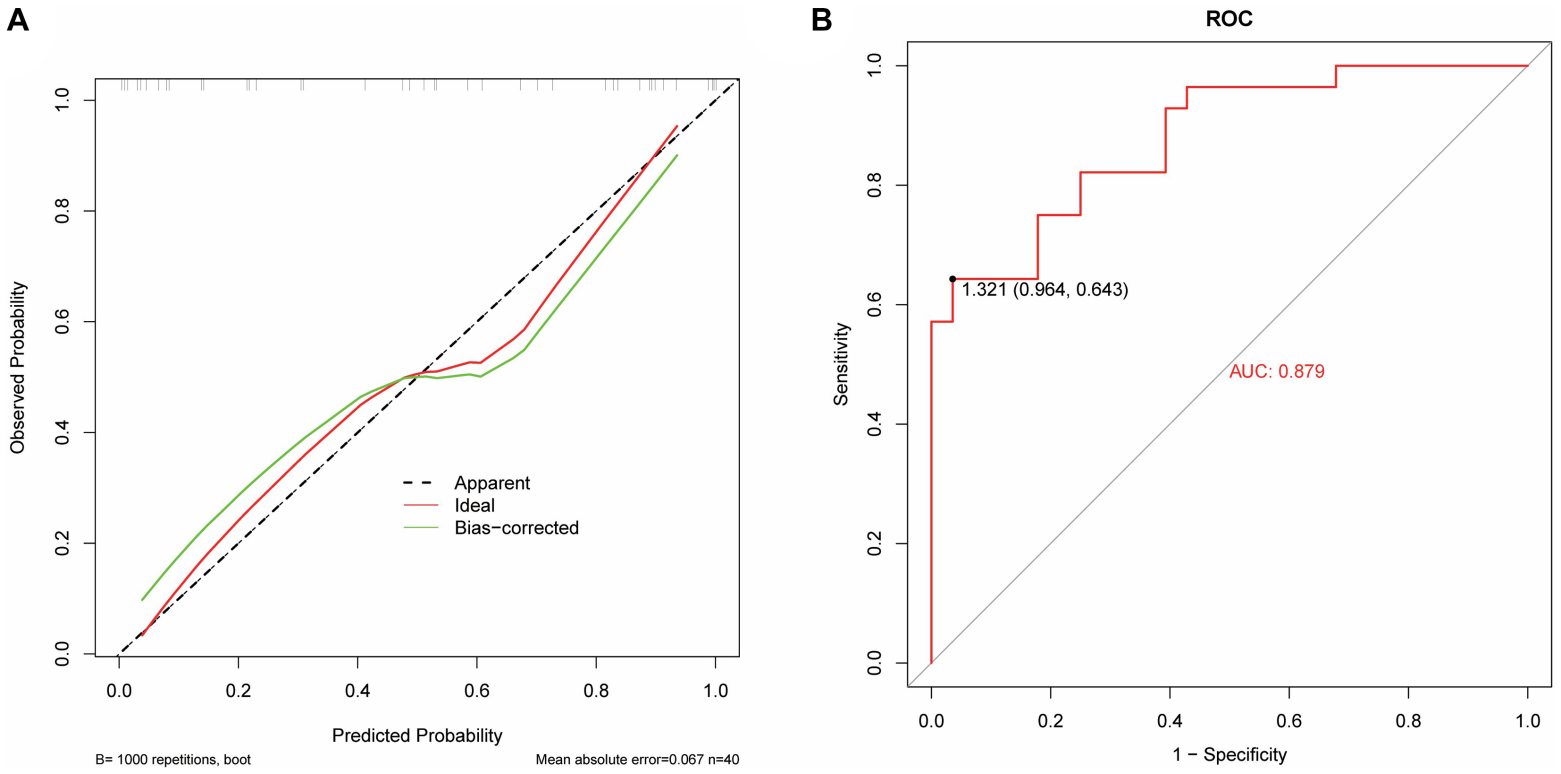
Performance evaluation of the LR model. **(A)** The calibrate calibration curves. **(B)** The ROC curve.

The model performance was evaluated and compared using the following seven metrics: AUC, sensitivity, specificity, accuracy, precision, recall (F1), and prAUC. In comparison, among all the machine learning models, the LR model performed the best in classification (AUC value was 0.897 in the training set), and the sensitivity, specificity, accuracy, precision, F1, and prAUC values were also the highest in the LR model (Table 1); therefore, the optimal model was LR. We visualized the LR model and plotted the nomogram for easy clinical application (Figure 6). A calibration curve was utilized to assess the model’s performance, and it can be seen that the error between the predicted values and the real values of the prediction model was small, and the result was highly accurate. The AUC = 0.879 for the model’s ROC curve, demonstrating that LR had the best predictive power (Figure 7).

**Table 1:** Model construction and evaluation.

Model	AUC	Sensitivity	Specificity	Accuracy	Precision	F1	prAUC
adaboost	0.659	0.635	0.630	0.632	0.665	0.695	0.622
GBDT	0.559	0.410	0.685	0.547	0.613	0.609	0.742
LR	0.897	0.800	0.850	0.825	0.842	0.820	0.776
NB	0.775	0.600	0.845	0.723	0.824	0.751	0.615
RF	0.801	0.670	0.670	0.670	0.711	0.716	0.602
SVM	0.785	0.645	0.655	0.650	0.692	0.713	0.620

## 4 Discussion

Sepsis is a serious infectious disease, and its pathogenesis involves multiple factors such as the immune system, inflammatory mediators, and vascular endothelium. The clinical manifestations of sepsis are diverse and it is necessary to comprehensively consider infection control, inflammation regulation, and organ support during treatment. Studies have shown that there may be potential benefits in the treatment and prevention of sepsis through the regulation of intestinal flora and the use of microbial preparations. The microbiota in the gastrointestinal tract of the human body is composed of trillions of bacteria that form the mucosal barrier of the intestine and are present in different proportions and numbers in different parts of the intestine, thus playing a defensive and protective role (11). Dysregulation of the microbiome or a reduction in microbial diversity is associated with altered immune responses. Sepsis affects the composition of the intestinal microbiome, which is characterized by loss of diversity, reduced abundance of key symbiotic bacteria such as *Faecium* and *Gastrococcus*, weakened colonization capacity of *Proteus* and other conditioning pathogens, and overpropagation, growing as a dominant bacterial group (12). A prospective cohort study of over 200 preterm infants in 2019 found that increased bacterial diversity and anaerobic colonization of the neonatal gut microbiome protected newborns from sepsis (13). The results of metagenomic sequencing technology in this study showed that the microbial abundance and diversity in patients with sepsis were significantly higher than those in the normal group (*p* < 0.05). The top 10 microbial abundances in the sepsis and normal groups were *Vitis vinifera*, *Mycobacterium canettii*, *Solanum pennellii*, *Ralstonia insidiosa*, *Ananas comosus*, *Moraxella osloensis*, *Escherichia coli*, *Staphylococcus hominis*, *Camelina sativa*, and *Cutibacterium acnes*, which mainly include protein families: genetic information processing, translation, protein families: signaling and cellular processes, and unclassified: genetic information processing.

Nowadays, traditional biomarkers such as CRP, PCT and IL-6 are widely used in the diagnosis and evaluation of sepsis (9). Inflammation serves as a defensive reaction aimed at eliminating invading pathogens, mitigating detrimental stimuli, and initiating tissue healing. The inflammatory response is activated when innate immune cells detect antigenic structures via pattern recognition receptors that identify molecular patterns associated with pathogens or damage-related molecular patterns (14). Excessive inflammatory response contributes to tissue damage and organ dysfunction in individuals with sepsis. Neutrophils produce reactive oxygen species through chemotaxis and phagocytosis, leading to widespread inflammation and increased microvascular permeability. Excessive inflammation causes a large number of neutrophil degranulation and proteolytic enzyme release, resulting in systemic and local endothelial damage (15). Therefore, neutrophils reflect the inflammatory response and immune status of the body during sepsis. IL-6 not only activates neutrophils but also delays phagocytosis of senescent and dysfunctional neutrophils, thereby exacerbating the production of post-traumatic inflammatory mediators and promoting the onset of post-traumatic systemic inflammatory response syndrome (16). Normal human serum IL-6 levels are very low, but when the body has an inflammatory response, IL-6 levels are significantly increased, and its level are increased earlier than other acute stage proteins, so it is helpful for the early diagnosis of emergency infection patients and can reflect the change in the disease condition (17). CRP is an acute phase protein produced by hepatocytes under the action of IL-6, and the serum CRP level in healthy people is very low; however, it can be significantly increased during bacterial infection, tissue damage, or stress, and it is significantly increased at the early stage of inflammation, which is a sensitive indicator of bacterial infection (18, 19). PCT is a hormone-free calcitonin peptide. Under normal physiological conditions, serum PCT levels are extremely low (20). However, under the action of inflammatory cytokines, the liver, kidneys, muscles, adipose tissue, and other solid organs of septic shock patients produce a large amount of PCT, resulting in a significant increase in the blood PCT levels of patients (20). Therefore, it can be used to diagnose, evaluate, and predict infectious diseases by measuring serum PCT levels in patients. Serum amyloid A (SAA) can be used as a sensitive indicator to reflect body infection and inflammation management, playing a crucial role in the adjunct diagnosis of infectious diseases (21). WBC is a common indicator of systemic inflammation, and relevant studies have reported that WBC count can diagnose early sepsis and is closely related to its prognosis (22). By analyzing the correlation between sepsis detection indicators and pathogenic microorganisms, it is possible to improve the diagnostic accuracy of sepsis, formulate more effective treatment options, and evaluate the prognosis of patients, which can help improve the recovery and survival rates of patients with sepsis. In this study, the correlation between sepsis-related inflammatory indicators, pathogenic microorganisms, and metabolic pathways was analyzed. The correlation analysis revealed a significant positive correlation (*p* < 0.05) between IL-6 and membrane transport. Metabolism of other amino acids showed a significant positive correlation (*p* < 0.05) with *Cutibacterium acnes*, *Ralstonia insidiosa*, *Moraxella osloensis*, and *Staphylococcus hominis*. *Ananas comosus* showed a significant positive correlation (*p* < 0.05) with poorly characterized and unclassified: metabolism. Blood test-related indicators showed a significant negative correlation (*p* < 0.05) with microorganisms.

Radiomics is the high-throughput extraction of a large amount of information from medical images to achieve lesion segmentation, feature extraction, and model establishment. It assists clinicians in making the most accurate diagnosis by conducting deeper mining, prediction, and analysis of massive amounts of image data information. It can also be intuitively understood as converting visual image information into deep quantitative features (23). In recent years, with the enhancement of computer data processing capabilities, improvement of image recognition technology, and continuous improvement of machine learning algorithms, in-depth data information of massive medical images can be mined and analyzed (24, 25). This capability has been applied to assess the severity of diseases (26), construct disease monitoring systems (automated alerting system) (27), and enable early prediction of diseases (28–31). Zhang et al. (32) found that the establishment of an XGBoost prediction model can predict sepsis-associated delirium earlier and is suitable for patients who are difficult to evaluate using traditional methods. Ge et al. (29) developed a machine learning model to accurately predict the occurrence of sepsis-associated acute brain injury and provide a basis for early intervention and treatment. In this study, the AUC value of the model based on the features extracted by radiomics was 0.791, the AUC of the medical record data features is 0.873; the AUC value of the logistic regression model based on the features extracted from medical records and radiomics was 0.879. It is proven that the model prediction ability is better when the two features are combined.

In this study, the blood samples of patients with sepsis were metagenomically sequenced to explore the complex relationship between microorganisms, metabolic pathways and blood test indicators, which provided a new idea and method for the diagnosis of sepsis. At the same time, a machine learning model based on medical records and radiomic features was developed for clinical diagnosis of sepsis, which filled some gaps in this field. The sample size of this study was small; the results may have been affected by sample selection, and further expansion of the sample size is required to verify the stability of the conclusions. Although the establishment of machine learning models has achieved certain prediction capabilities, they still need to be verified and optimized on larger datasets.

## 5 Conclusion

Taken together, microbial abundance and diversity were elevated in the sepsis group. Correlation analysis of blood test-related indicators with microbial and metabolic pathways showed significant correlations, which might contribute to further clinical diagnosis and treatment. The LR prediction model based on radiomics and medical record data had good diagnostic efficacy and calibration for identifying patients with sepsis, which is a potential auxiliary tool for clinical decision-making.

## Data Availability

The original contributions presented in the study are included in the article/supplementary material, further inquiries can be directed to the corresponding author.
